# Engineered Porosity ZnO Sensor Enriched with Oxygen Vacancies Enabled Extraordinary Sub-ppm Sensing of Hydrogen Sulfide and Nitrogen Dioxide Air Pollution Gases at Low Temperature in Air

**DOI:** 10.3390/s24237694

**Published:** 2024-11-30

**Authors:** Engin Ciftyurek, Zheshen Li, Klaus Schierbaum

**Affiliations:** 1 Department of Materials Science, Institute for Experimental Condensed Matter Physics, Heinrich Heine University of Düsseldorf, 40225 Düsseldorf, Germany; klaus.schierbaum@uni-duesseldorf.de; 2 ASTRID2 Synchrotron Light Source, ISA, Centre for Storage Ring Facilities, Department of Physics and Astronomy, Aarhus University, Ny Munkegade 120, 8000C Aarhus, Denmark; zsli@phys.au.dk

**Keywords:** hydrogen sulfide (H_2_S), nitrogen dioxide (NO_2_), sensor, oxygen vacancy, adsorbed oxygen, XPS, tortuosity, AES, SOFCs, electrical resistance, surface chemistry, zinc oxide

## Abstract

We report the results of a zinc oxide (ZnO) low-power microsensor for sub-ppm detection of NO_2_ and H_2_S in air at 200 °C. NO_2_ emission is predominantly produced by the combustion processes of fossil fuels, while coal-fired power plants are the main emitter of H_2_S. Fossil fuels (oil, natural gas, and coal) combined contained 74% of USA energy production in 2023. It is foreseeable that the energy industry will utilize fossil-based fuels more in the ensuing decades despite the severe climate crises. Precise NO_2_ and H_2_S sensors will contribute to reducing the detrimental effect of the hazardous emission gases, in addition to the optimization of the combustion processes for higher output. The fossil fuel industry and solid-oxide fuel cells (SOFCs) are exceptional examples of energy conversion–production technologies that will profit from advances in H_2_S and NO_2_ sensors. Porosity and surface activity of metal oxide semiconductor (MOS)-based sensors are both vital for sensing at low temperatures. Oxygen vacancies ($V_{O}^{••}$) act as surface active sites for target gases, while porosity enables target gases to come in contact with a larger MOS area for sensing. We were able to create an open porosity network throughout the ZnO microstructure and simultaneously achieve an abundance of oxygen vacancies by using a heat treatment procedure. Surface chemistry and oxygen vacancy content in ZnO were examined using XPS and AES. SEM was used to understand the morphology of the unique characteristics of distinctive grain growth during heat treatment. Electrical resistivity measurements were completed. The valance band was examined by UPS. The Engineered Porosity approach allowed the entire ZnO to act as an open surface together with the creation of abundant oxygen vacancies ($V_{O}^{••}$). NO_2_ detection is challenging since both oxygen (O_2_) and NO_2_ are oxidizing gases, and they coexist in combustion environments. *Engineered porosity ZnO* microsensor detected sub-ppm NO_2_ under O_2_ interference, which affects mimicking realistic sensor operation conditions. *Engineered porosity ZnO* performed better than the previous literature findings for H_2_S and NO_2_ detection. The exceptionally high sensor response is attributed to the *high number of oxygen vacancies (*$V_{O}^{••}$*)* and *porosity extending through the thickness of the ZnO with a high degree of tortuosity*. These features enhance gas adsorption and diffusion via porosity, leading to high sensor response.

## 1. Introduction

Fossil fuel usage has risen dramatically, about eightfold since the 1950s and approximately doubling since the 1980s. Fossil fuels have been fundamental to building industrialized societies worldwide and accumulating wealth. In contrast, their detrimental effects on the environment, human health, and the climate suggest that we must move away from them. Despite obviously the worst climate crises across the globe affecting everyday life severely, it is apparent that the energy industry will utilize fossil-based fuels more in the coming decade [1].

We have witnessed a record utilization of a wide range of fossil-based energy sources after the industrial revolution. In 2019, the production output of oil and petroleum reached a record high. Similarly, natural gas plant liquid (NGPL) production has been rising steadily since 2005 and reached a record high in 2023. Renewable energy output in 2023 was about 9%. The contribution of coal to total U.S. energy output has declined from 37% in 1950 to 9% in 2023, equalizing it to the total renewable contribution in the same year. The anticipated upsurge in the accumulation of hazardous emissions (H_2_S and NO_2_) into the environment places greater importance on the environmental and operational concerns associated with hydrocarbon management and energy production–conversion in the near term. The major energy production in the USA by source is as follows: natural gas 36%, oil 38%, followed by coal 9%. Fossil fuels (oil, natural gas, and coal) retained 78% of energy production in 2017, which increased to 83% in 2024, while renewable energy is only 9% of the total output [2,3,4].

One of the main drivers of the gas sensor market’s rapid growth is the need to monitor environmentally hazardous gases [5]. Gas sensors for pollutants and toxic gases such as nitrogen dioxide (NO_2_) and hydrogen sulfide (H_2_S) receive significant attention to safeguard human health, nature, and habitat [6,7]. These sensors are also used to optimize various energy production–conversion and regulate exhaust emissions and desulphurization [8]. NO_2_ and H_2_S are principal air pollutants that cause harm to ecosystem balance, destroying the stratosphere’s protective ozone layer and accelerating deforestation. NO_2_ is easily oxidized to nitric acids. H_2_S is also infamously known to be used as a chemical warfare agent during WW2 [9,10,11,12]. The primary and secondary exposure limits for NO_2_ is 1 ppm arithmetic mean annual concentration, while it is 5 ppm for H_2_S [13].

NO_2_ and H_2_S emissions are primarily produced by the combustion of natural gas, oil, and coal in refineries, process furnaces, fluid catalytic cracking (FCC) regenerators, electric power plant boilers, and gas turbines, where they are converted into serviceable products for energy production–conversion for commercial and residential use [14,15,16]. Overall, 65% of total NO_2_ emission is produced by human activity stemming from combustion processes such as cars, ships, airplanes, petroleum refineries, industrial processes, and power plants. Handling H_2_S and NO_2_ is challenging at every phase of hydrocarbon, including production, purifying, and transport. Emission control has become a major challenge due to the increased utilization of fossil fuels by both developing and developed nations [17,18,19,20,21,22].

Solid oxide fuel cells (SOFCs) are promising in the energy production conversion industry due to their low-carbon footprint without hazardous exhaust emissions, such as H_2_S and NO_2_ [23]. SOFCs can utilize a variety of renewable, environmentally friendly, and inexpensive fuel sources, such as natural and landfill gases, coal syngas, biomass, sewage, and municipal and green wastes. Considering the composition of the fuels, the emission of the following hazardous gases is anticipated: NO_2_ and H_2_S.

The Ni-based (nickel oxide) composite is the most common anode material for SOFCs; however, the just-mentioned gases found in fuel sources cause degradation of SOFCs by poisoning the Ni-based anode. As a result, the energy conversion–production industry, as well as green energy production initiatives, will greatly benefit from real-time monitoring of H_2_S and NO_2_ concentration to regulate/maintain optimal fuel utilization. In addition to that, safe-guarding environmental protection will be in place without expensive and cumbersome regulations–filtering–testing requirements [17,18,24,25].

*H_2_S detection* is realized by the following MOS-based gas sensors: WO_3_, CeO_2_, SnO_2_, ZnO, CuO, platinum and palladium oxides, Fe_2_O_3_, In_2_O_3_, TiO_2_, and CdO [5,26,27,28,29]. WO_3_ and SnO_2_ lacked stability and showed cross-sensitivity, in addition to a requirement for high temperature (>300 °C). Table 1 provides the bulk of the X-ray electron spectroscopy (XSP) and Auger electron spectroscopy (AES) reported literature, along with our findings for ZnO. Table 2 provides the literature findings for photoelectron based analysis H_2_S sensors, materials, and performances, together with results obtained in our current work. Liewhiran et al. used SnO_2_ for H_2_S sensing at 300 °C; the sensor response (S) was 8 for 10 ppm H_2_S [30]. Some mixed–modified unconventional materials were investigated for H_2_S at lower temperatures. Fe_2_O_3_-Fe_2_(MoO_4_)_3_ showed high sensitivity for H_2_S at 225 °C. La_0.7_Pb_0.3_Fe_0.4_Ni_0.6_O_3_ and CdIn_2_O_4_ did not exhibit sufficient sensing at a temperature range of 100–350 °C. Varying ferrites were also tried, but the magnitude of sensitivity was in the range of 0–10% resistivity change between 100–400 °C [31,32,33,34]. Kersen utilized Fe_2_O_3_ with Fe_2_(MoO_4_)_3_ thick oxide films for 1, 10, and 20 ppm of H_2_S at 225 °C [5,34]. Sun et al. utilized carbon nanotube templated hematite (α-Fe_2_O_3_) against 10 ppm H_2_S at 130 °C [29]. Chaudhari et al. concluded that TiO_2_ with 5 wt.% Al_2_O_3_ and 0.5 wt% Pd increased the sensor response (S) up to 0.8 at 250 °C for 200–1000 ppm of H_2_S, while beyond 350 °C, a step decrease was detected in sensor response [35,36]. Ciftyurek et al. attributed this detrimental decrease to the abrupt desorption of chemisorbed oxygen species after 350 °C by showing a sudden drop in the amount of chemisorbed oxygen species by using photoelectron techniques [37,38]. Shirsta et al. reported on polyaniline nanowires decorated with gold nanoparticles for the detection of H_2_S at 25 °C [39].

*NO_2_ detection* materialized mainly through ZnO, WO_3_, TiO_2_, SnO_2_, In_2_O_3_, V_2_O_5,_ and NiO. Numerous synthesis/deposition approaches, such as sputtering, spray pyrolysis, atomic layer deposition (ALD), chemical vapor deposition (CVD), physical vapor deposition (PVD), sol–gel, spray pyrolysis, and hydrothermal technique, have been utilized to produce ZnO sensor in pure or doped–alloyed forms within various multi-component heterostructures for NO_2_ sensing. Cai et al. used gold (Au)-doped ZnO nanowires activated via ultraviolet light (UV) that produced a 2.3 sensor response to 1 ppm of NO_2_ 25 °C [40]. ZnO equipped with reduced graphene oxide (rGO) detected NO_2_ at 110 °C [41]. Doping ZnO with Ni showed a 108% increase in electrical resistance against 100 ppm NO_2_ at 200 °C [42]. Au–polyaniline/ZnO nanocomposite showed a sensor response of 21 against 50 ppm of NO_2_ at 300 °C [43]. Table 2 provides the literature findings for H_2_S and NO_2_ sensors, materials, and performances, together with results obtained in our current work. Mai et al. reported on ALD deposited 50 nm thick ZnO sensor for NO_2_ sensing at 330 °C without any oxygen interference, but the sensor response was fairly low due to the confined and densely packed microstructure of ZnO and lower concentration of oxygen vacancies ($V_{O}^{••}$) [44]. In other words, this low sensor signal is the outcome of a lack of porosity–tortuosity due to the jam-packed grain structure on the ZnO surface. This densely packed microstructure decreases both the effective ZnO surface area that comes in contact with NO_2_ and target gas penetration through the thickness of the ZnO.

ZnO thin films have been a dynamic field of research and application due to their applications in electronics, biomedicine, sensing applications, transducers, optoelectronics, and catalysts since the 1960s. ZnO is a direct, wide-band gap metal oxide semiconductor (MOS). The most standard utilization form of ZnO is its polycrystalline form. ZnO has a direct band gap energy of 3.37 eV. ZnO has a hexagonal lattice and is made up of two interconnecting sublattices of Zn^2+^ and O^2−^ in wurtzite crystal symmetry such that each O^2−^ ion is enveloped by tetrahedra of Zn^2+^ ions. This architecture’s large piezoelectric and pyroelectric properties result from missing the center of symmetry [45].

Metal oxide semiconductor (MOS)-based sensors are the most frequently used gas sensors due to their straightforward operation principle with high sensitivity and compatibility with inexpensive mass fabrication approaches such as screen printing and MEMS. Conversely, MOS sensors suffer from high energy consumption due to high working temperature, which is required for the generation–activation of chemisorbed oxygen species. The oxygen species are essential for gas sensing in metal oxide-based sensors. Adsorption of molecular oxygen via chemisorption and dissociation onto stoichiometric metal oxide surfaces is not possible, while both are readily feasible on non-stoichiometric metal oxide surfaces [37,38,46].

In our current work, we selected ZnO due to its easiness to integrate desired surface physical and chemical features at lower temperatures. By design, we are aiming to have proper surface chemistry promotion to accommodate a high amount of chemisorbed oxygen species, in addition to the desired surface texture–porosity facilitating gas diffusion through the porosity network open to the external surface. The creation of more active oxygen species, such as (${O^{2-},O}^{-},O_{2}^{2-},O_{2}^{-}$), is favorably associated with the ZnO surface activity. Oxygen vacancy sites ($V_{O}^{••}$) function as dissociative centers to convert oxygen molecules into desired oxygen species (${O^{2-},O}^{-},O_{2}^{2-},O_{2}^{-}$).

We remarkably improved the sensor response of ZnO at lower temperatures using defect chemistry and sintering principles. By creating an open microstructure for gas diffusion and oxygen vacancy abundance on the ZnO surface, we were able to increase the effective surface area and tortuosity of ZnO and enhance the catalytic sensing reactions required for sensing to be realized at lower temperatures. In this work, we created oxygen vacancy–abundant high porosity ZnO by heat treatment, leading to controlled coarsening of ZnO nanosized grains under the low-temperature sintering conditions applied.

The gas-sensing mechanism and sequence were explained based on electrical and surface chemistry measurements. The microstructural characterization was completed through SEM analysis. Surface chemistry of ZnO and stoichiometry analysis for the oxygen vacancy ($V_{O}^{••}$) concentration were realized through AES, XPS, and UPS measurements. NO_2_ detecting in combustion environments, such as automobile exhaust, is challenging because of the coexistence of O_2_ and NO_2_ in combustion processes; this is because we utilized oxygen (O_2_) gas together with NO_2_ throughout the sensor tests to realize realistic testing conditions.

The H_2_S and NO_2_ detection using MOS at low temperatures with high sensor response without an expensive catalyst and/or other cumbersome modification techniques is very challenging. In our work, we developed the Engineered Porosity approach, leading ZnO with high porosity and tortuosity together with abundant oxygen vacancies ($V_{O}^{••}$) that enabled NO_2_ and H_2_S sensing at 200 °C with an extraordinarily high sensor response. We developed a micro gas sensor architecture based on *Engineered Porosity ZnO* with high tortuosity and abundant oxygen vacancies.

## 2. Experimental

**Figure 1:**
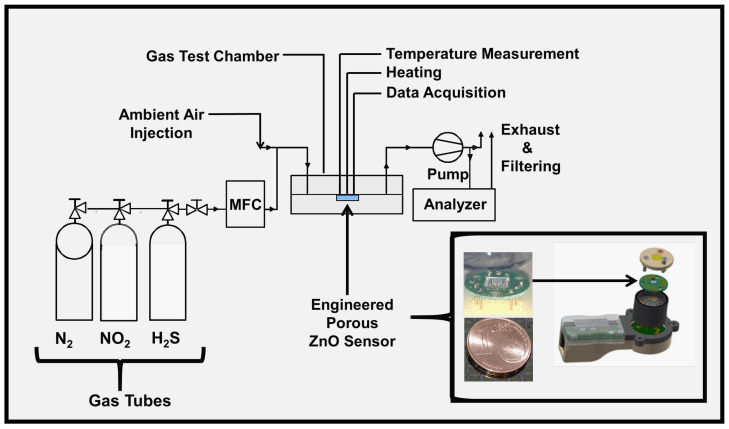
The schematic view of gas testing components and peripheral units used in H_2_S and NO_2_ testing. The integrated sensor architecture includes a heating element, a temperature sensor, and *the Engineered Porosity ZnO* sensing layer.

The ZnO was deposited over sapphire (Al_2_O_3_) substrates at 30 nm thickness. The sensor head shown in Figure 1 contains an integrated platinum (Pt) heating element and a Pt-1000 heat sensor. The ZnO was characterized by vdP, XPS, UPS, AFM, and SEM. An in-house designed 4-point probe was used for van der Pauw (vdP) electrical resistivity measurements between 25–350 °C. The data acquisition was completed with National Instruments (NI, Austin, TX, USA) PXIe-1071 digital multimeter (DMM). The XPS and UPS investigations were accomplished using Material Science Beamline (MATLINE) at the ASTRID2 synchrotron facility in Aarhus University, Aarhus, Denmark. The spectra for all elements were referenced to Au 4f_7/2_ at 84.00 eV, and Fermi level corrections were applied. The samples were not sputter cleaned before analysis to avoid misguiding results, as it is well known that sputter cleaning causes reduction of metal oxides or preferential sputtering of surface adsorbed species. The sensor tests were carried out with H_2_S and NO_2_. The sensor response (S) for reducing gas H_2_S is defined as the ratio of R_air_/R_gas_, while the response for oxidizing gas NO_2_ is defined as R_gas_/R_air_, in which R_gas_ is the electrical resistance of ZnO sensor in the air, while R_gas_ is electrical resistance upon exposure to reducing (H_2_S) or oxidizing (NO_2_) gas. Figure 1 shows the details of the sensor architecture and gas testing setup. The test gases were mixed from the ultra-high purity compressed gas bottles using mass flow controllers. For H_2_S testing, 0.2 ppm to 2.25 ppm H_2_S pulses were diluted in high-purity nitrogen (NO_2_) carrier gas. NO_2_ tests were carried out between 3 ppm and 15 ppm NO_2_ carried in N_2_ under intentional interference of 4.5 to 18 ppm oxygen (O_2_). For H_2_S and NO_2_, 30 and 15 min pulses were realized.

## 3. Result

### 3.1. Conception and Creation of Engineered Porosity ZnO with Oxygen Vacancies ($V_{O}^{••}$)

ALD makes use of low deposition temperature for metal oxides, resulting in fine grain size with low surface roughness and limited porosity, which all severely reduces surface area. Because of their limited porosity, metal oxides produced by the ALD technique have hardly any gas adsorption sites in the as-deposited state. From the perspective of gas sensor design, the adsorption of oxygen species (${O^{2-},O}^{-},O_{2}^{2-},O_{2}^{-}$) on MOS is crucial for gas sensing action. The sensing performance of the MOS sensor can be improved by generating more gas adsorption sites, creating more oxygen vacancies ($V_{O}^{••}$), and boosting the surface catalytic activity.

These objectives can be accomplished in a variety of ways, including surface alloying, nanocomposite formation, ion bombardment, precious metals additives, and nanosize texturing. In order to create limited grain coarsening and grain growth on the ZnO surface that increases the open porosity network, surface area, and vacancy abundance, we used a unique heat treatment technique in our work.

Grain-to-grain contact and coarsening can reduce the total surface area and minimize the thin film’s total interfacial and surface energy [18,47,48,49,50,51,52,53,54,55,56,57]. This work will primarily concentrate on the development of coarsening and grain growth through the distinct control of sufficient grain-to-grain contact with a high-level porosity network through the ZnO film thickness. We created *Engineered Porosity ZnO Enriched with Oxygen Vacancies* to have inter-grain contacts without extensive necking, thus having a large porosity network with vast tortuosity. Fine homogeneously distributed and interconnected porosity established throughout the material is crucial for target gas distribution pathways and essential to expanding the contact area between sensing material ZnO and porosity interface.

### 3.2. ZnO Microstructure Analysis

**Figure 2:**
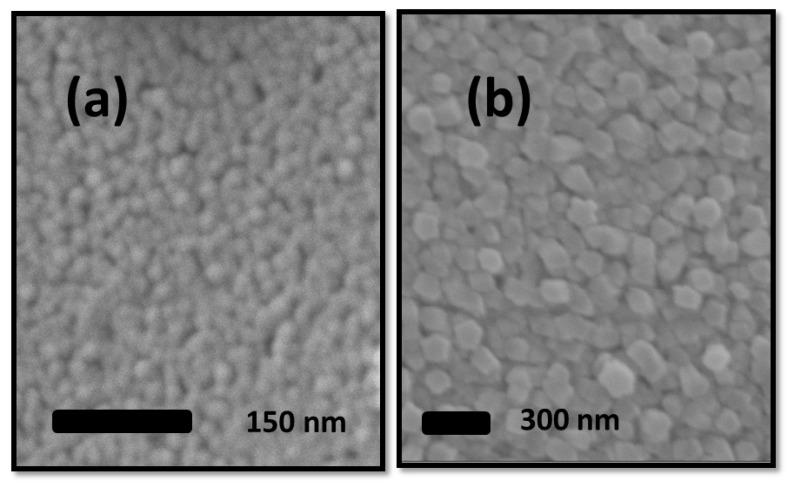
The 30 nm thick ZnO; SEM micrograph (**a**) before heat treatment and (**b**) after heat treatment at 300 °C for 4 h.

ZnO was deposited to a thickness of ~30 nm, with a grain size of ~15 nm. Figure 2a shows the as-deposited state of the ZnO; a poorly percolated ZnO microstructure maintains low tortuosity throughout the polycrystalline thin film. Low-level grain coalescence is the result of granular microstructure produced by low-temperature sputtering and evaporation thin film deposition techniques. The thin films having nanosize grains maintain a high driving force for grain growth and coarsening.

As shown in Figure 2b, a vermicular-shaped ZnO microstructure with an average grain size of ~100 nm was produced in our work following 4 h of annealing at 300 °C. The SEM micrograph also demonstrates how the annealing of ZnO formed a structure that included more porosity, tortuosity, and clearly visible ZnO grains. In contrast to materials with larger grain sizes, the thermodynamic stabilization of surface area minimization is the driving force behind the unique features of grain growth/coarsening during the sintering of nanograin-sized ZnO.

High-level gas adsorption sites and a fine and homogeneous granular structure are achieved. Heat treatment that resulted in grain coarsening expanded the gas diffusion pathways greatly. It is crucial to note that ZnO coarsening resulted from grain boundary migration and that the grain boundaries did not impede the grain coarsening because they also co-occurred with the reduction–induced creation of oxygen vacancies.

A high degree of tortuosity resulted from the controlled sintering–coarsening process, which simultaneously increased porosity and grain boundaries. Section 3 will go into detail about the chemistry of gas adsorption sites. The heat treatment led to the abundance of oxygen vacancy concentration on the surface, together with well-defined gas diffusion pathways with high tortuosity. Both produce ideally perfect gas sensing conditions.

### 3.3. Zinc Oxide (ZnO) Electrical Resistance Investigation

Normally, ZnO maintains a small amount of oxygen deficiency. This makes transition metal oxide ZnO an oxygen-deficient n-type semiconductor with a typical electrical resistivity of 0.01–3 Ω·m at room temperature [58,59,60,61]. The substantial deviances among the reported electrical resistivity values in the literature are linked to diverse deposition systems and/or post-deposition treatment, resulting in different microstructures, stoichiometries, and surface properties. Oxygen deficiency, or in other words, oxygen vacancies, has a strong effect on the electrical, chemical, and sensory properties of the ZnO. The oxygen vacancies and oxygen deficiency were investigated and examined using UPS and XPS in the Section 3.4 and Section 3.5.

**Figure 3:**
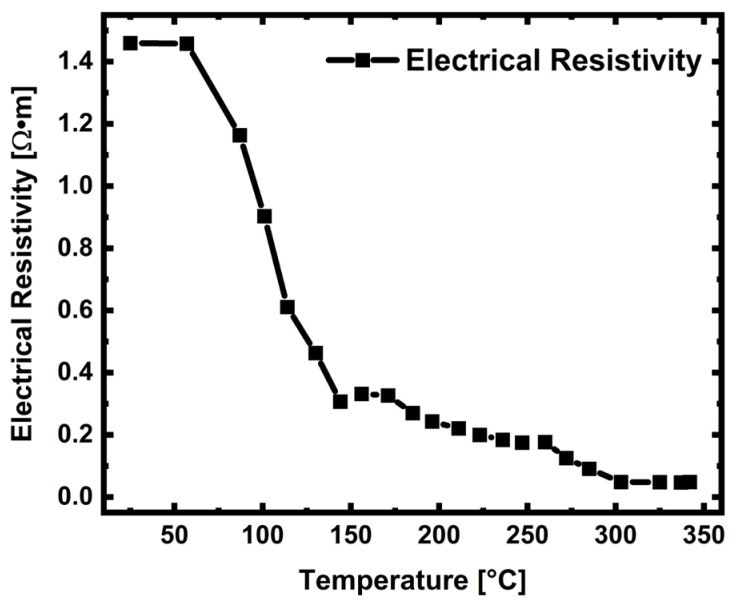
Electrical resistivity measurement for 30 nm thick ZnO film from 25 °C to 350 °C.

Figure 3 displays the resistivity values of the 50 nm thick ZnO thin film. The electrical resistivity as a function of temperature was measured under environmental conditions in the 25–350 °C temperature range. An increase in temperature continually reduced the resistivity values for the ZnO thin film. The resistivity at room temperature (25 °C) was 1.45 Ω·m, and it showed a sharp decrease down to 0.30 Ω·m at 140 °C. When the temperature was raised to 150 °C, the resistivity decreased and rhythm slowed. Due to lattice uptake of adsorbed oxygen, some of the oxygen vacancies ($V_{O}^{••})$ on the surface were annealed, causing a slight decrease after 200 °C.

### 3.4. ZnO Valence Band Investigation

The Zn 4s valance electrons form a bond with the oxygen (O) 2p valance electrons in ZnO, where Zn ions are in tetrahedral coordination with O ions. The bonding between Zn and O ions is ionic, owing to the large variance in their respective electronegativities, as Zn possesses 1.65, while O has 3.44. We measured the ZnO valance band with 150 and 300 eV excitation X-ray photons to probe the surface (0.5 nm depth from the surface) and subsurface areas (1 nm depth from the surface) independently.

**Figure 4:**
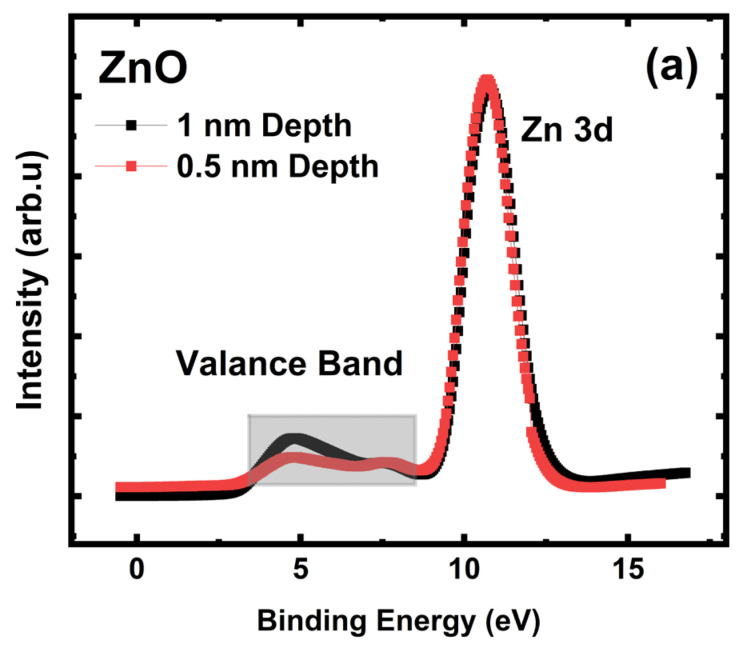

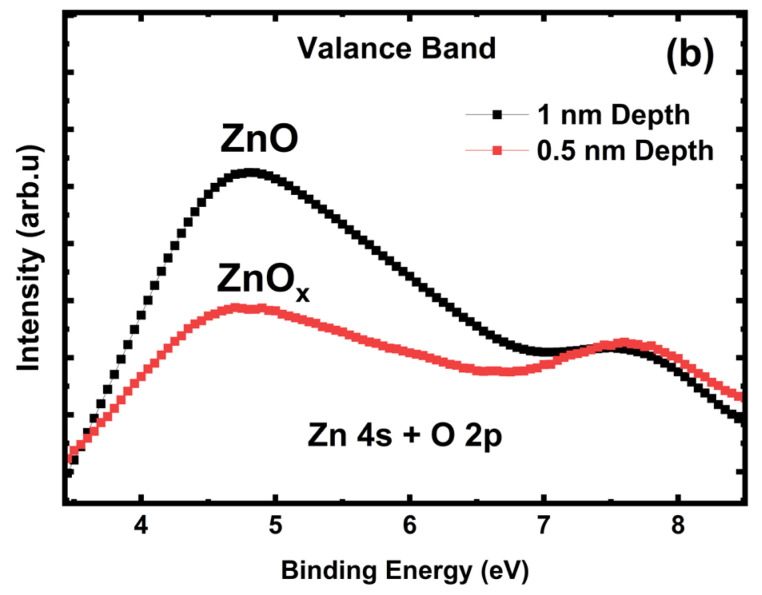
*Engineered porosity ZnO*; 1 nm and 0.5 nm depths from the surface. (**a**) Valance band and Zn 3d regions together. (**b**) Enlarged view of the valence band region; Zn 4s + O 2p blending feature.

Figure 4 shows the valance band region of ZnO in the vicinity of the Zn 3d peak. Figure 4a shows Zn 3d and the valance band region together with distinct bordering. The valence band is represented by Zn 4s-O 2p blended states near 3–8.5 eV, while Zn 3d is observable near 10.7 eV. The broad features of the Zn 4s-O 2p valance band suggest that surface stoichiometry is highly influenced by the high amount of oxygen vacancies ($V_{O}^{••})$**.**

At 1 nm depth measurement, the valance band shows defect-free features of fully stoichiometric ZnO. A decrease in the occupation of the Zn 4s + O 2p blended states from 1 nm to 0.5 nm depth measurements indicates an increase in the concentration of surface oxygen vacancies. At 0.5 nm measurement depth, oxygen vacancies ($V_{O}^{••})$ were observed in the valance region as they gave rise to a perturbation around 7–8 eV at the same time, causing a decrease in the intensity around the 4–7 eV region.

As seen in Figure 4b, the deeper measurement (1 nm depth, black-colored) confirmed the stoichiometric ZnO phase. However, the 0.5 nm (red-colored) depth measurement showed the dominant existence of oxygen vacancy ($V_{O}^{••})$-rich ZnO_x_ phase on the surface. The intensity of Zn 4s + O 2p blended state decreased from ZnO to ZnO_x_, thus showing the outer surface is dominated by the reduced—oxygen deficient —oxygen vacancy rich ZnO_x_ phase.

### 3.5. ZnO Auger Peak (AES) Analysis for Stoichiometric Understanding

AES has larger chemical shifts than XPS core-level shifts, so it allows chemical state analysis in cases that are unlikely for XPS [59]. Determination of the oxidation state of zinc (Zn) will contain significant uncertainties if it is solely based on XPS analysis of zinc (Zn) 2p photoelectron line. In the best scenario, Zn 2p demonstrates a minor shift of ~0.2 eV in the binding energy between zinc (Zn) metal and fully oxidized ZnO; in most cases, there is a strong overlap [62]. The binding energy for Zn 2p_3/2_ for Zn metal and ZnO are 1021.7 eV and 1021.9 eV, respectively [63,64]. Due to this slight shift in fully oxidized ZnO and Zn metal, performing quantitative–qualitative peak analysis and determining the oxidation state of Zn-based XPS 2p analysis is very challenging. To overcome this obstacle, we proposed to utilize AES in conjunction with XPS.

In situations where XPS is unable to identify the chemical state, a shift of about 4 eV in the Auger peaks distinguishes Zn metal from ZnO. AES is an effective surface-sensitive technique capable of probing down to 1 nm depth below the surface. Using the Auger line L_3_M_45_M_45_, a precise chemical state analysis of ZnO was performed. AES peaks are historically represented in the electron kinetic energy scale [65]. Figure 2 shows L_3_M_45_M_45_ position for ZnO. According to published reports in the literature, L_3_M_45_M_45_ values for ZnO range from 987 eV to 989 eV. In contrast, L_3_M_45_M_45_ for metallic Zn ranges from 992 eV to 993 eV. Table 1 provides the bulk of the XSP and AES reported literature, along with our findings for ZnO.

**Figure 5:**
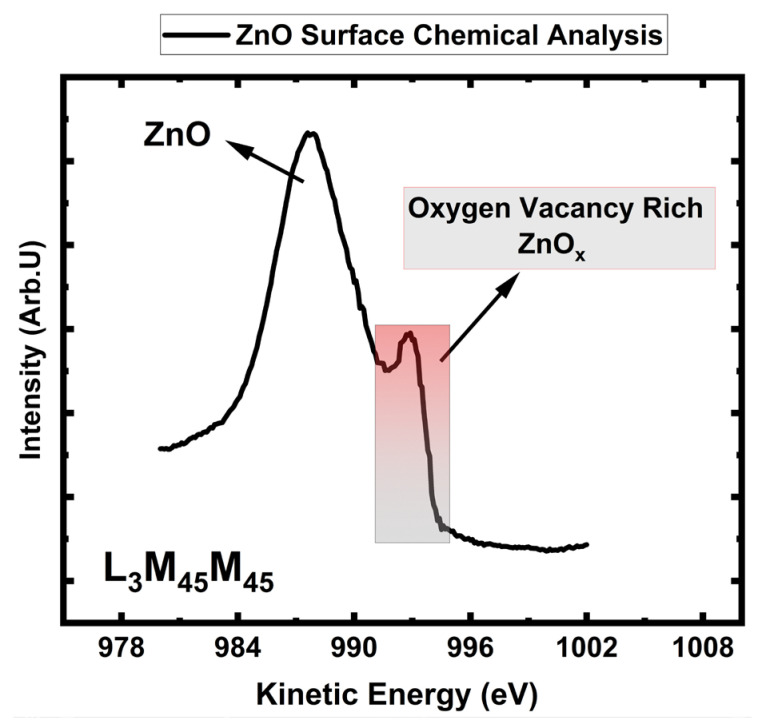
ZnO L_3_M_45_M_45_ position with 1147 eV excitation X-ray photons.

We report 987.9 eV for zinc oxide (ZnO) L_3_M_45_M_45_. On the other hand, in Figure 5, we propose that the shoulder seen on the higher kinetic energy site located at 992.3 eV presents ZnO_x_. ZnO_x_ phase contains large amounts of oxygen vacancies, and this is because it showed up in a similar region that metal Zn reported in the literature. Thanks to a larger kinetic energy separation between ZnO and ZnO_x_, the shoulder strongly confirmed the existence of oxygen vacancy ($V_{O}^{••})$-rich zinc oxide (ZnO_x_) as a minority phase within the measurement depth of 1 nm. The amount of the corresponding phases will be calculated from the XPS measurements presented in the following section. We showed that the outer ZnO surface is covered by the oxygen vacancy-rich ZnO_x_ phase.

**Table 1:** The core-level XPS and AES literature findings for ZnO in comparison with our measurement results.

Electron Level	Zn 3s in ZnO	Zn 3p_1/2_ in ZnO	Zn 3p_3/2_ in ZnO	Zn 3d in ZnO	O 1s in ZnO	L_3_M_4,5_M_4,5_ in ZnO	L_3_M_4,5_M_4,5_ in Zn
Ciftyurek and Schierbaum (this work)	140.0	91.6	88.8	10.7	529.8	987.9	992.3
Vesely and Langer [66]	139.8	92.0	89.0	10.5	530.9	988.9	-
Gaarenstroom and Winograd [67]	-	-	-	10.7	-	987.7	-
Kowalczyk [68]	-	-	-	-	-	-	991.9
Wagner [69]	-	-	-	-	-	-	992.0
Schoen [65]	139.6	91.8	88.7	10.3	530.3	988.5	992.5
Barr and Hackenberg [70]	-	-	-	10.3	530.3	987.9	992.0
Klein and Hercules [71]	-	-	-	10.4	-	988.2	992.3
Strohmeier and Hercules [72]	139.2	-	88.3	-	529.9	988.9	992.4
Powell [73]	-	-	-	-	-	-	992.4
Ley and Kowalczyk [74]	-	-	-	10.4	-	-	991.9
Wehner and Mercer [75]	-	-	-	-	-	988.1	992.1
Dake and Baer [76]	-	-	-	-	-	988.1	992.2

### 3.6. Concentration of Oxygen Vacancies ($V_{O}^{••})$ in Engineered Porosity ZnO

Oxygen vacancies facilitate replenishment of the adsorbed oxygen (${O^{2-},O}^{-},O_{2}^{2-},O_{2}^{-}$) on the ZnO sensor surface, which is essential for chemical sensing in metal oxides. Therefore, it is important to measure the concentration of the oxygen vacancies ($V_{O}^{••})$. Concentration of oxygen vacancies can be calculated either from the oxidation state of the zinc (Zn) in the ZnO or oxygen (O) 1s peak analysis. In our current work, we analyzed both Zn and O via XPS in *engineered porosity ZnO enriched with oxygen vacancies.*

#### 3.6.1. Zn 3p Analysis

**Figure 6:**
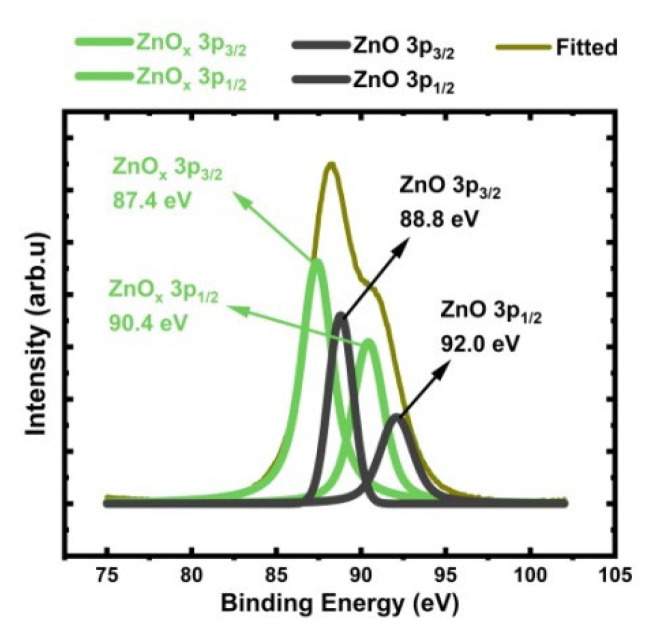
Deconvoluted Zn 3p peak, showing non-stoichiometric ZnO_x_ associated with oxygen vacancies.

Auger analysis proved the existence of the ZnO_x_ phase on the ZnO surface. We continued with Zn 3p peak analysis to determine the amounts of each ZnO and ZnO_x_. The Zn 3p spectrum from the depth of 1 nm is shown in Figure 6. Deconvolution analysis was used to quantify the amounts of ZnO and oxygen vacancy ($V_{O}^{••})$ abundant non-stoichiometric ZnO_x_. The Zn 3p envelope was deconvoluted into two distinct chemical states: stoichiometric ZnO and ZnO_x_. We measured the binding energy of 3p_3/2_ for ZnO_x_ as 87.4 eV, while 3p_3/2_ for ZnO is 88.8 eV. The binding energies (BE) we reported for 3p_3/2_ for ZnO and ZnO_x_ are in good agreement with the literature values [65,66,72,77]. The decrease in the 3p electron binding energy in ZnO_x_ compared to the ZnO is due to the high level of oxygen vacancies created in the ZnO_x_, as it was processed through heat treatment–sintering–coarsening for creating porosity and tortuosity.

The amounts for ZnO and ZnO_x_ phases are 33 at.% and 67 at.%, respectively. ZnO_x_ is a dominant phase and is homogeneously distributed through the surface, dictating sensor interaction with the target gaseous environment.

#### 3.6.2. Oxygen 1s Analysis

**Figure 7:**
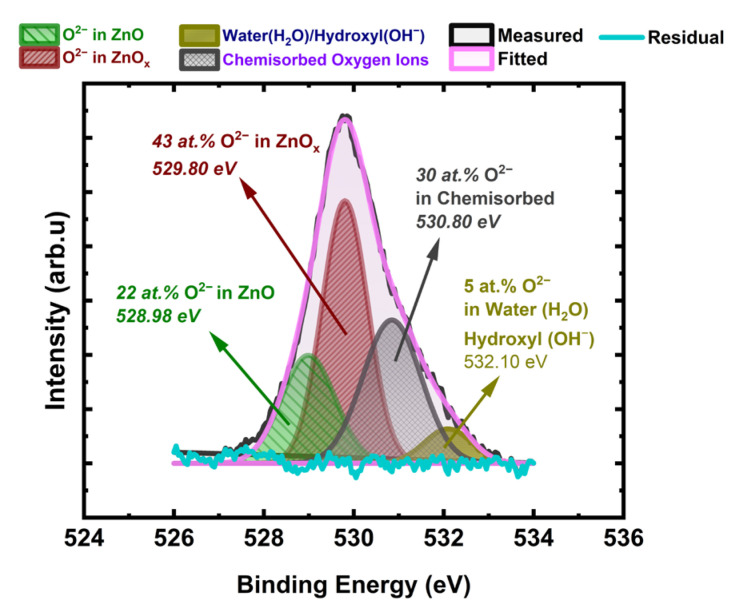
O 1s spectrum with 545 eV excitation photon energies corresponding to 1 nm depth from the Engineered Porosity *ZnO* sensor surface. Binding energies and amount of each component are also provided in the graph.

Figure 7 shows the XPS spectrum of oxygen O 1s spectrum from the Engineered Porosity *ZnO* surface. The quantification of different oxygen ion-containing species, such as water/hydroxide groups (H_2_O/OH^−^), chemisorbed oxygen ions (${O^{2-},O}^{-},O_{2}^{2-},O_{2}^{-}$), and lattice oxygen ions connected to ZnO_x_ and ZnO, was completed. The analysis also focused on the calculation amounts of ZnO and ZnO_x_ separately. The O 1s spectra are fitted to four sub-spectra centered at 528.98, 529.80, 530.80, and 532.10 eV, accounting for the lattice oxygen ions in ZnO, lattice oxygen ions in oxygen vacancy-rich ZnO_x_, chemisorbed oxygen ions, and water/hydroxyl groups, respectively. The binding energies we report for water/hydroxide, chemisorbed oxygen, and lattice oxygen ions are in good agreement with the literature reported for ZnO and other metal oxides used for gas sensing [18,37,38,78,79].

The amount of chemisorbed oxygen ions and water/hydroxyl groups are 30 at.% and 5 at.%, respectively. The high amount of chemisorbed oxygen ions shows that ZnO_x_ is rich in oxygen vacancies since the chemisorbed oxygen ions attach oxygen vacancy ($V_{O}^{•}$ and $V_{O}^{••}$) centers on the ZnO surface. The amount of lattice oxygen ions connected to ZnO is 22 at.%. However, the amount of lattice oxygen ions in oxygen vacancy-rich ZnO_x_ is 43 at.%. That shows that the majority of the ZnO sensor surface interactions are dictated by the oxygen vacancy-abundant ZnO_x_.

#### 3.6.3. Zinc (Zn) to Oxygen (O) Stoichiometric Quantification (ZnO)

We used intensity factors and the ratio between the normalized areas of the O 1s and Zn 3p contributions; O/Zn was found to be 0.73 at.%. We determined x in ZnO_x_, x = 0.73, so the oxygen vacancy abundant phase is ZnO_0.73_. That shows that the majority of the ZnO sensor surface is governed by the oxygen vacancy abundant ZnO_0.73_, which we named throughout paper as ZnO_x_

### 3.7. Gas Sensor Testing for H_2_S and NO_2_

#### 3.7.1. An Overview of the Sensing Mechanism and the Involvement of Oxygen Ions in Metal Oxide Semiconductor (MOS) Sensors’ Gas Sensing Reactions

The sensing mechanism for all MOS sensors is explained on the basis of the adsorption/chemisorption of oxygen ions on the MOS surface. The concentration of a certain specific type of negatively charged adsorbed/chemisorbed oxygen ions dictates the number of electrons in the MOS sensor conduction band, thus establishing a constant electrical resistance (R). ${O^{2-},O}^{-},O_{2}^{2-},O_{2}^{-}$ are the most common chemisorbed oxygen species taking place in gas sensing reactions [37,38,80,81,82,83,84,85].

Equations (1) and (2) illustrate the adsorption and dissociative adsorption of oxygen gas and consumption of conduction band electrons from ZnO, leading to an increase in electrical resistance (R). Subsequently, adsorbed oxygen ions dissociated on the ZnO surface through the processes governed by Equation (3) through Equation (5), requiring the consumption of additional ZnO electrons and raising the electrical resistance even further. Equation (6) shows the annihilation of the oxygen vacancy site via adsorption and dissociation of oxygen molecules into vacancy location under suitable temperature and oxygen partial pressure conditions. Target gases, such as H_2_S, CO, SO_2_, etc., consume the adsorbed oxygen ions, thus leading to the return of electrons to the ZnO conduction band, decreasing the electrical resistance [18,48,80,81,82,84,85,86]. Equations presented in Equations (1)–(6) are reversible if certain conditions are satisfied.

(1) $$O_{2(gas)}+e^{-}\to O_{2(adsorbed)}^{-}$$

(2) $$O_{2(gas)}+2e^{-}\to 2O_{\left(adsorbed\right)}^{-}$$

(3) $$O_{2(adsorbed)}^{-}+e^{-}\to O_{2(adsorbed)}^{2-}$$

(4) $$O_{2(adsorbed)}^{2-}\to 2O_{\left(adsorbed\right)}^{-}$$

(5) $$2O_{\left(adsorbed\right)}^{-}\to O_{\left(adsorbed\right)}^{2-}$$

(6) $$O_{\left(adsorbed\right)}^{2-}+V_{o}^{••}\to \;O_{O}^{\times }$$

Monoatomic oxygen ions (${O^{2-},O}^{-}$) are more reactive compared to molecular oxygen ions ($O_{2}^{2-},O_{2}^{-}$) [37,38]. The creation of $\;O_{2}^{-}$ (Equation (1)) through initial adsorption on metal oxide surface is an exothermic reaction that results in a ~1 eV decrease in free energy, whereas other reactions presented in Equations (2) through (6) are endothermic. $O_{2}^{-}$ transformation to $O^{-}$ occurs at ~150–200 °C (Equations (3)and (4)). The dissociation of $O_{2}$ into $O^{-}$ (Equations (2) and (4)) necessitates ~0.5 eV. Monoatomic oxygen ions (${O^{2-},O}^{-}$) can tolerate temperatures up to >400 °C before departing the ZnO surface. $O^{2-}$ ions comparing to $O_{2}^{2-},O_{2}^{-}$, and $O^{-}$ are more long-lasting at elevated temperatures on a ZnO surface; moreover, if $O^{2-}$ ions are trapped (Equation (6)) by $V_{O}^{•}$ and $V_{O}^{••}$ vacancy centers, $O^{2-}$ ions cannot be distinguished from the lattice oxygen ions. The creation of $O_{2}^{2-}$ (Equation (3)) requires about 5 eV, and a formation of $O^{2-}$ (Equation (5)) will require higher energy of ~20 eV [37,38,81,82,87,88].

The most active and populous species of adsorbed oxygen ions are anticipated to be $O^{-}$ at the testing temperature of 200 °C used in this work. This is because we will only include $O^{-}$ in the sensing mechanism equations from Equations (7)–(12) that are presented in Kröger–Vink notation.

#### 3.7.2. Initial Tests on Adsorption Kinetics with Oxygen (O_2_) and Nitrogen (N_2_)

**Figure 8:**
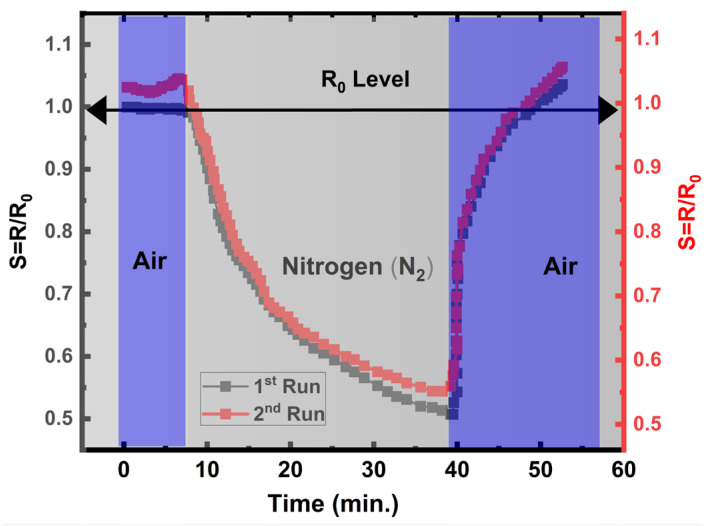
Adsorption and dissociation of O_2_ molecules on ZnO and their effect on the electrical resistance at 200 °C in air. High purity N_2_ introduced between air pulses containing 21% O_2_.

We investigated the absorption–dissociation of O_2_ on the Engineered Porosity *ZnO* surface. The O_2_ source was atmospheric air. Figure 8 illustrates how the electrical resistance (R/R_0_) of *engineered porosity ZnO* changes upon exposure to O_2_ and N_2_ successively. R_0_ denotes electrical resistance, as the ZnO sensor is maintained under constant O_2_ flow, while R denotes the electrical resistance under N_2_ flow. N_2_ molecules kick off oxygen ions (${O^{2-},O}^{-},O_{2}^{2-},O_{2}^{-})$ from their positions on the surface as soon as O_2_ flow is eliminated by N_2_ introduction. This causes an injection of electrons back into the ZnO conduction band, which causes a ~55% decrease in the electrical resistance (R). Following the removal of the N_2_ flow, as shown in Figure 8 at the 40th minute, and the reintroduction of O_2_, the O_2_ adsorption–dissociation described in Equations (1)–(6) caused the Engineered Porosity *ZnO* electrical resistance to rise to the initial level.

*Engineered porosity ZnO* has a great deal of oxygen vacancies and a high degree of network of open porosity. Figure 8 illustrates how a single exposure to atmospheric air containing 21% O_2_ increased electrical resistance by nearly 100%. In other words, a large number of oxygen vacancies and high-level connected porosity facilitated a large amount of O_2_ adsorption and dissociation through the reactions given in Equations (1) through (6).

#### 3.7.3. Hydrogen Sulfide (H_2_S) Testing

**Figure 9:**
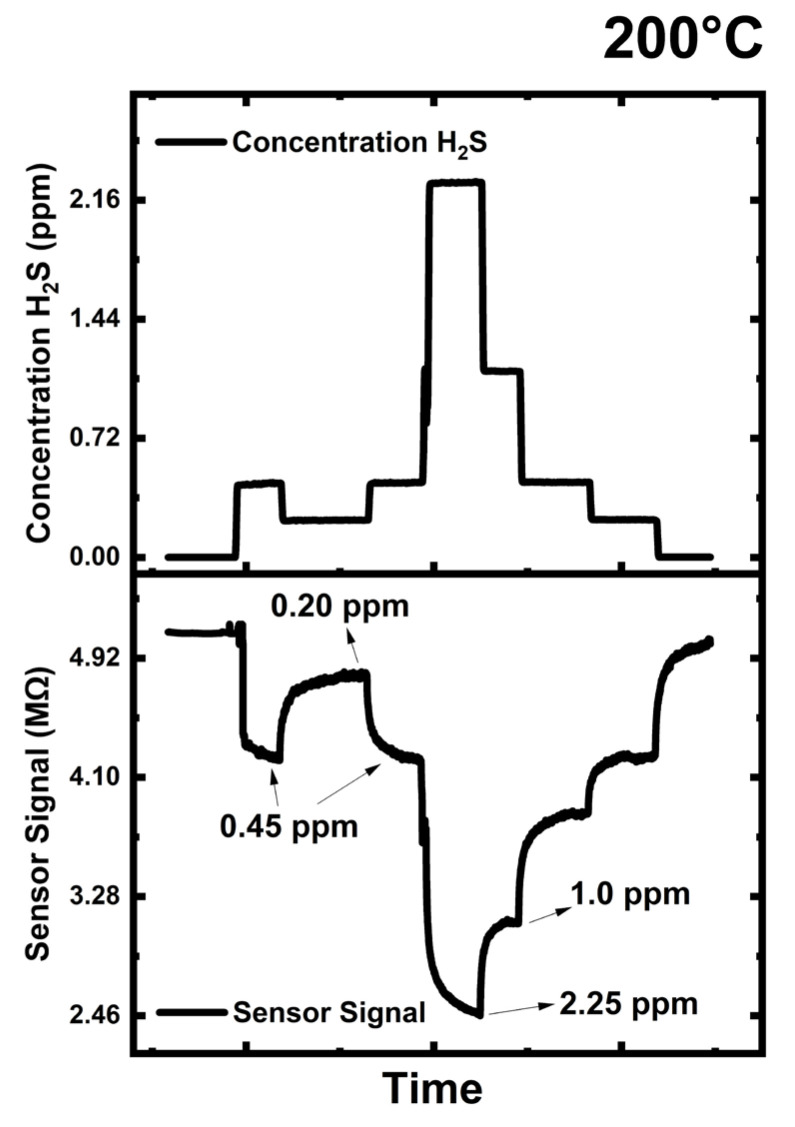
*Engineered porosity ZnO* tested for sub-ppm H_2_S at 200 °C.

Figure 9 shows the Engineered Porosity *ZnO* sensor’s H_2_S response at 200 °C as the concentration of H_2_S varies from a minimum of 0.2 ppm to a maximum of 2.25 ppm. The sensor showed typical n-type semiconductor performance by displaying a decrease in electrical resistance upon exposure to the H_2_S-reducing gas. During H_2_S exposures, chemisorbed oxygen ions interacted with H_2_S, releasing electrons back to the conduction band, thus decreasing the electrical resistance according to the mechanisms governed by Equations (7) and (8).

Our sensor testing temperature is 200 °C; thus, we consider $O^{-}$ to be the most active adsorbed oxygen specie because $O^{-}$ ion formation is favored at ~150–200 °C either by direct reduction from **O_2_** (Equation (2)) and/or transformation from $O_{2}^{-}\;$ to $O^{-}$ (Equations (3) and (4)). Equation (7) presents the reaction between H_2_S and $O^{-}$. As was previously mentioned, surface oxygen vacancy ($V_{O}^{••}$) defect centers associated with ZnO_x_ helped to facilitate the entire process presented in Equation (7). The reaction between H_2_S and adsorbed oxygen ($O^{-}$) produces the by-product of SO_2_ [17,18,46,81]. Because SO_2_ itself is also reducing agent, SO_2_ will interact with the adsorbed oxygen species in the manner outlined in Equation (8).

(7) $$H_{2}S_{\left(gas\right)}+{3O}_{\left(adsorbed\right)}^{-}\to SO_{2(gas)}+H_{2}O+3e^{-}$$

(8) $$SO_{2(gas)}+O_{\left(adsorbed\right)}^{-}\to SO_{3(gas)}+e^{-}$$

At the end of the H_2_S testing, the Engineered Porosity *ZnO* sensor reintroduced into an oxygen-rich atmosphere relocated the consumed adsorbed oxygen species in accordance with Equations (1)–(5); as a result, we observed the electrical resistance recovered back to its initial value before H_2_S introduction. SO_2_ is a by-product of the complete combustion of H_2_S. According to Equation (7), H_2_S reaction with adsorbed oxygen $O^{-}$ ion produces three free electrons (e^−^) per reaction, whereas SO_2_ reaction yields one free electron (e^−^). The sensing sequence for H_2_S ends with sulfur trioxide (SO_3_) as SO_2_ oxidizes to SO_3_, as seen in Equation (8).

In summary, *engineered porosity ZnO* abundant with oxygen vacancies showed an n-type sensing behavior for H_2_S. The ZnO sensor demonstrated exceptional sensing performance, exhibiting a high response to 1 ppm H_2_S with a 45% decrease in electrical resistance, yielding a sensor response of 1.7. Table 2 provides the literature findings for H_2_S with the various materials, compositions, dopants, precious metals, additions, etc., along with the results we obtained in our current work.

Examining Table 2, it is evident that *engineered porosity ZnO* has distinguished itself with exceptional properties and outperformed the majority of the tabulated literature thanks to its high sensor response and straightforward, simple, and affordable production method. Given that the most recent research in the literature, as shown in Table 2, makes use of costly precious metal addition, surface alloying, multi-step doping, and/or laborious nanocomposites, the ZnO sensor can differentiate between extremely low concentrations of H_2_S, including 2.25, 1, 0.45, and 0.2 ppm with high accuracy. The ZnO sensor, when exposed to CO_2,_ did not show any sensor response; it showed much less toward CO and that was less than a 1% change in electrical resistance.

#### 3.7.4. Nitrogen Oxide (NO_2_) Testing

**Figure 10:**
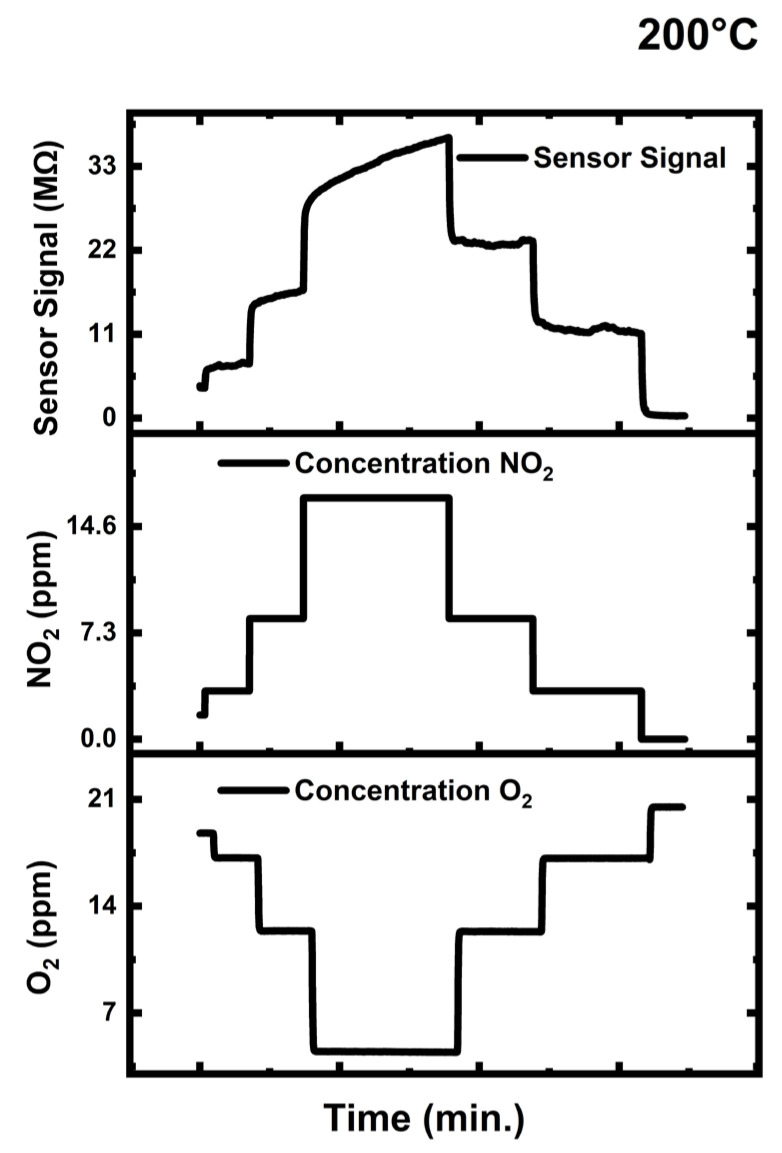
*Engineered porosity ZnO* tested for NO_2_ at 200 °C under O_2_ interference.

Figure 10 displays the testing results for NO_2_ at 200 °C, which also includes the concentrations profiles of O_2_ and NO_2_ during sensor testing. Combustion processes produce NO_2_, which, in fact, coexist in the same environment as another oxidation gas, namely oxygen (O_2_). Because of this demanding condition, *engineered porosity ZnO* was tested for NO_2_ while being interfered with by oxygen (O_2_). Even with high O_2_ interference, ZnO demonstrated exceptional sensing performance at different NO_2_ concentrations. Table 2 compares the NO_2_ sensor results from our current work with those from other studies that used different materials–compositions–dopants–precious metal additions and/or burden–some nanocomposite formations, etc., with our results obtained in the current work.

NO_2_ introduction increased the electrical resistance due to the consumption of conduction band electrons, as noted from Equations (9)–(12). Figure 10 illustrates how the sensor’s electrical resistance increased as the NO_2_ concentration was gradually raised, eventually reaching its saturation peak value for 15 ppm of NO_2_. As NO_2_ concentration decreased, the Engineered Porosity *ZnO* electrical resistance decreased to its initial value, emphasizing excellent reversibility and the sensor’s reliability and repeatability.

The Engineered Porosity *ZnO* sensor showed maximum response of 15 to 15 ppm NO_2_. Even under the interference of 18 ppm of O_2_, the sensor managed to detect 3 ppm of NO_2_. In comparison to the previous research provided in Table 2, the exceptional sensor response from *engineered porosity ZnO* is attributed to a straightforward development process, high porosity, well-established tortuosity in microstructure, and a large number of NO_2_ pull centers, specifically oxygen vacancies ($V_{O}^{••}$). The gas sensing tests of the Engineered Porosity *ZnO* sensor with CO_2_, CO, toluene, and H_2_ did not lead to detectable changes in the electrical resistance, so the results are not presented here.

The reactions for NO_2_ sensing are given from Equations (9)–(12). *Engineered porosity ZnO* has the capability to sense NO_2_ even under the presence of O_2_, as demonstrated by Equations (10)–(12). NO_2_ molecules can be adsorbed on oxidized sites on the ZnO_x_ surface (Equations (9) and (10)); on the other hand, the dissociation and adsorption of NO_2_ happen to be at oxygen vacancy sites ($V_{O}^{••}$), as seen in Equations (11) and (12) [38]. Dissociation and adsorption of NO_2_ are enabled by the surface oxygen vacancies abundantly found in *engineered porosity ZnO.* NO_2_ dissociates to NO by oxidizing surface oxygen vacancies via the donation of $O^{-}$ and $O^{2-}$ ions, as given in Equations (11) and (12).

(9) $$NO_{2(gas)}+e_{\left(surface\right)}^{-}\to NO_{2\;(adsorbed)}^{-}$$

(10) $$NO_{2(gas)}+{2e}_{\left(surface\right)}^{-}+O_{2(adsorbed)}^{-}\to NO_{2(adsorbed)}^{-}+2O_{\left(adsorbed\right)}^{-}$$

(11) $$NO_{2(gas)}\overset{\left(adsorption\right)}{\to }V_{O}^{••}+2e_{\left(surface\right)}^{-}\to (V_{O}^{••}-O_{\left(adsorbed\right)}^{2-})+NO_{\left(gas\right)}$$

(12) $$O_{2(gas)}+ZnO_{x}\overset{\left(adsorption\right)}{\to }\left(ZnO_{x}-NO_{2(adosrbed)}^{-}\right)\overset{\left(desoprtion\right)}{\to }(ZnO_{x}-O_{\left(gas\right)}^{-})+{NO}_{\left(gas\right)})$$

As an interference gas, different concentrations of oxygen (O_2_) were added. This was completed in order to determine whether changes in oxygen gas concentrations could have an impact on the gas-sensing mechanism in situations like those found in typical combustion environments with high and fluctuating oxygen content. Because oxygen (O_2_) interference cannot credibly impact the sensor signal, as demonstrated by the experiments, *the Engineered Porosity ZnO* is extraordinarily effective at detecting NO_2_ in realistic environmental settings.

Table 2 reviews H_2_S and NO_2_ sensing performances of different compositional and microstructural formations of ZnO, including nanofiber, nanocomposites, nanorods, and nanospheres in various architectures to improve the sensor response. CuO, SnO_2_, WS_2,_ and some of the materials were used in addition to the costly precious metals such as platinum (Pt), gold (Au), and palladium (Pd) as surface decoration, doping, additives, and/or mechanical alloying. In addition to being difficult, costly, and time-consuming to manufacture, these compositional and microstructural formations have limited applicability for large-scale practice within an acceptable standardization for real-world industrial use. *Engineered porosity ZnO* enriched with surface oxygen vacancies ($V_{O}^{••}$) showed a sensor response of 15 towards 15 ppm of NO_2_ and 2.2 to 2.25 ppm of H_2_S 200 °C. *Engineered porosity ZnO* produced very successful sensor results for NO_2_ and H_2_S sensing when compared to the sensor results from complex doped–alloyed composite material sets presented in Table 2.

*Engineered porosity ZnO* did not have any sensing promoter agents commonly used in the other sensing materials presented in Table 2. This work uses engineered porosity-designed surface oxygen vacancy ($V_{O}^{••}$)-enriched ZnO, which *outperforms other sensor* platforms–configurations (see Table 2) in terms of *sensor response magnitude, simplicity, and design robustness. In our work, we reached such an extraordinary sensor response for pure ZnO material thanks to the heat treatment, engineered porosity–tortuosity network, and enriched surface oxygen vacancy concentration.*

**Table 2:** Gas sensing results obtained in this work for engineered porosity ZnO, together with the literature findings for H_2_S and NO_2_ under different gas concentrations and testing temperatures. Variety of dopants and addition of other oxide composites and/or precious metals modified ZnO literature findings tabulated for the comparison purposes.

Sensing Material Composition and Physical State and Form	Sensor Testing Temperature (°C)	Test Gas Concentration	Response Magnitude (R_air_/R_gas_) for H_2_S (R_gas_/R_air_) for NO_2_
**Engineered porosity ZnO (this work)**	**200**	**2.25 ppm H_2_S**	**2.2 (this work)**
CuO/SnO_2_–ZnO core shell NWs [89]	25	10 ppm H_2_S	1.6
ZnO/SnO_2_ nanofibers [90]	250	50 ppm H_2_S	63.3
ZnO nanorods [91]	250	10 ppm H_2_S	20
Cu–ZnO nanograins [92]	250	15 ppm H_2_S	0.9
Au-modified ZnO nanowires [93]	25	5 ppm H_2_S	6.1
Pd–SnO_2_–ZnO [94]	25	20 ppm H_2_S	0.06
ZnO nanowires [95]	25	1 ppm H_2_S	1.5
**Engineered porosity ZnO (this work)**	**200**	**15 ppm NO_2_**	**15 (this work)**
ZnO thin film [96]	250	1 ppm NO_2_	2.4
ZnO/SnO_2_-rGO nanocomposite [97]	30	5 ppm NO_2_	1.4
UV-irradiated Au-doped ZnO [40]	25	1 ppm NO_2_	2
Co-doped ZnO nanocapsules [98]	280	100 ppm NO_2_	3.86
Ni-doped ZnO [42]	200	100 ppm NO_2_	2
Au-decorated ZnO-PANI [43]	300	50 ppm NO_2_	14

## 4. Conclusions

We created a microsensor architecture for the low temperature (200 °C) detection of environmentally hazardous gases (NO_2_ and H_2_S) in the air using zinc oxide (ZnO) enriched with oxygen vacancy ($V_{O}^{••}$) population and a high degree of interconnected porosity and tortuosity. We established a heat treatment procedure leading to controlled nanograin coarsening in order to produce *engineered porosity ZnO with* a network of porosity and tortuosity throughout the active layer thickness.

The sensor demonstrated sensor response against 2.25 ppm of H_2_S with a response of 2.2, while for 15 ppm of NO_2_, the response was 15 at 200 °C. *Engineered porosity* ZnO showed an exceptionally high response at low temperatures with excellent stability–reversibility–repeatability through quick response–recovery times in comparison to the literature findings tabulated in Table 2. This high sensor response is attributed to two primary reasons: firstly, an abundance of oxygen vacancies ($V_{O}^{••}$) created by the heat treatment procedure, and secondly, novel sintered/coarsened grain formation that activated a network of porosity and island-like connected structures.

Surface analysis of *engineered porosity ZnO* revealed that lattice oxygen attached to ZnO is 22 at.%, whereas 30 at.% of oxygen is found in chemisorbed oxygen. Lattice oxygen in the oxygen vacancy-rich phase ZnO_x_, on the other hand, is 43 at.%. This indicates that the oxygen vacancy-abundant ZnO_x_ controls the majority of the ZnO sensor surface interactions. The O/Zn was found to be 0.73 at.%. We determined x in ZnO_x_, x = 0.73, so the oxygen vacancy-abundant phase is ZnO_0.73_.

*Engineered porosity ZnO* detected sub-ppm NO_2_ even under oxygen (O_2_) interference, affecting the simulation of actual sensor operating conditions. The application we accomplished is feasible for industrial-level gas sensor production thanks to the straightforward creation of an engineered porosity ZnO sensing layer on the sensor chip illustrated in Figure 1.

In conclusion, the *heat treatment–coarsening–sintering* strategy for the creation of oxygen vacancies designed to simultaneously form connected open porous microstructure through the ZnO film led to the exceptional gas sensing properties for NO_2_ and H_2_S at 200 °C. Surface oxygen vacancies assisted in the capture of target gases and oxygen ions, which propel the sensing to extraordinarily high levels because of the partially empty d-bands achieved in ZnO_x_.

## Data Availability

The original contributions presented in this study are included in the article material. Further inquiries can be directed to the corresponding author.
